# Association Between Meeting Physical Activity Guidelines with Ambulation and Quality of Life in Claudication

**DOI:** 10.1093/geroni/igab046.2548

**Published:** 2021-12-17

**Authors:** Andrew Gardner, Polly Montgomery, Ming Wang, Biyi Shen, Shangming Zhang, William Pomilla

**Affiliations:** 1 Penn State College of Medicine, Hummelstown, Pennsylvania, United States; 2 Penn State College of Medicine, Penn State College of Medicine, Pennsylvania, United States

## Abstract

We determined if meeting the 2018 physical activity guidelines was associated with better ambulatory function, health-related quality of life, and inflammation than failing to meet the guidelines in patients with peripheral artery disease and claudication. Secondly, we determined the optimal number of total daily steps that are needed to meet the physical activity guidelines. Five hundred seventy-two patients were assessed on their daily ambulatory activity for one week with a step activity monitor, and were grouped according to whether they achieved less than 150 minutes of moderate intensity physical activity per week (Group 1=Do Not Meet Guidelines; n=397), or whether they were above this threshold (Group 2=Meet Guidelines; n=175). Treadmill peak walking time (mean±SD) was significantly higher (p<0.001) in Group 2 (709±359 sec) than in Group 1 (427±281 sec). The health-related quality of life score for physical function was significantly higher (p<0.001) in Group 2 (61±22%) than in Group 1 (44±21%). High sensitivity C-reactive protein was significantly lower (p<0.001) in Group 2 (3.6±4.5 mg/L) than in Group 1 (5.9±6.1 mg/L). Finally, 7,675 daily steps was the optimal threshold associated with meeting the physical activity guidelines, with a sensitivity of 82.9% and a specificity of 88.4%. In conclusion, patients with claudication who meet the 2018 physical activity guidelines for US adults had better ambulation, HRQoL, and inflammation outcomes than those who failed to meet the guidelines. From a practical standpoint, patients with claudication best achieved the physical activity guidelines by taking a total of 7,675 daily steps.

